# Effective flow-through polishing strategies for knob-into-hole bispecific antibodies

**DOI:** 10.1186/s40643-022-00590-8

**Published:** 2022-09-14

**Authors:** Serene W. Chen, Kong Meng Hoi, Farouq Bin Mahfut, Yuansheng Yang, Wei Zhang

**Affiliations:** 1 Downstream Processing Group, Bioprocessing Technology Institute, Agency for Science, Technology and Research, Singapore, Singapore; 2 Cell Line Development Group, Bioprocessing Technology Institute, Agency for Science, Technology and Research, Singapore, Singapore

**Keywords:** Bispecific antibody, Knob-into-hole, Flow-through polishing, Host cell proteins, High molecular weight impurities, Low molecular weight impurities, Higher aggregation propensity

## Abstract

Bispecific antibodies (bsAbs), though possessing great therapeutic potential, are extremely challenging to obtain at high purity within a limited number of scalable downstream processing steps. Complementary to Protein A chromatography, polishing strategies play a critical role at removing the remaining high molecular weight (HMW) and low molecular weight (LMW) species, as well as host cell proteins (HCP) in order to achieve a final product of high purity. Here, we demonstrate using two knob-into-hole (KiH) bsAb constructs that two flow-through polishing steps utilising Capto Butyl ImpRes and Capto adhere resins, performed after an optimal Protein A affinity chromatography step can further reduce the HCP by 17- to 35-fold as well as HMW and LMW species with respect to monomer by ~ 4–6% and ~ 1%, respectively, to meet therapeutical requirement at 30–60 mg/mL-resin (R) load. This complete flow-through polishing strategy, guided by Design of Experiments (DoE), eliminates undesirable aggregation problems associated with the higher aggregation propensity of scFv containing bsAbs that may occur in the bind and elute mode, offering an improved ease of overall process operation without additional elution buffer preparation and consumption, thus aligning well with process intensification efforts. Overall, we demonstrate that through the employment of (1) Protein A chromatography step and (2) flow-through polishing steps, a final product containing < 1% HMW species, < 1% LMW species and < 100 ppm HCP can be obtained with an overall process recovery of 56–87%.

**Graphical Abstract:**

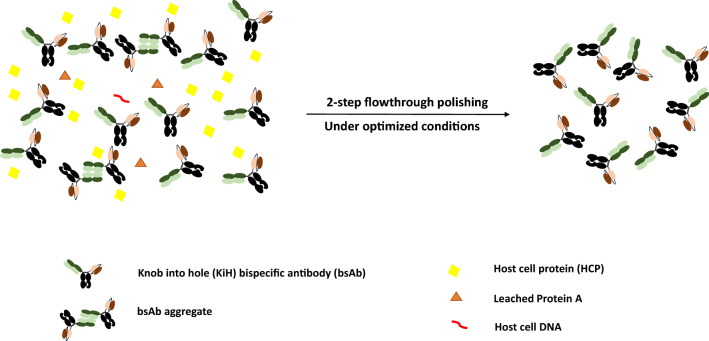

## Introduction

Bispecific antibodies (bsAbs) represent a particularly promising class of biotherapeutics due to their ability to bind to two different antigens, opening up a wide range of potential therapeutic applications (Kontermann 2005; Baeuerle and Reinhardt 2009; Chames and Baty, 2009; Kontermann 2012; Brinkmann and Kontermann 2017; Labrijn et al. 2019; Gaber, 2014). This is exemplified by the four FDA approved bsAbs currently in the market, namely blinatumomab, emicizumab, amivantamab and faricimab, along with a wide variety of reported bsAb formats and large numbers in clinical development (Kontermann 2005; Baeuerle and Reinhardt 2009; Chames and Baty, 2009; Kontermann 2012; Brinkmann and Kontermann 2017; Labrijn et al. 2019; Gaber, 2014; Gökbuge et al. 2018; Kantarjian et al. 2017; Oldenburg et al. 2017; Neijssen et al. 2021; Syed 2021). Development of effective strategies for their downstream processing is therefore important to keep in step with these breakthroughs in upstream development, preventing any potential bottleneck in their subsequent industrial manufacturing process as conventional downstream processing methods may be ineffective at the removal of specific product and process-related impurities associated with this important class of biotherapeutics.

In particular, bsAbs are often associated with a higher level of impurities and byproducts, including aggregates, fragments and mispaired products (Garber 2014; Taki et al. 2015; Andrade et al. 2019; Michaelson et al. 2009; Michaelson et al. 2009; Jakobsen et al. 2011, Klein et al. 2012), with the generally lower titers of bsAbs often translating to relatively higher host cell protein (HCP) levels. We (Chen et al. 2022), along with others (Tustian et al. 2016, 2018; Lindhofer et al. 1995; Smith et al. 2015; Zwolak et al. 2017a, b; Zwolak et al. 2017a, b; Skegro, et al. 2017; Ollier et al. 2019; Chen et al. 2020; Zhang et al. 2021), have reported the effective use of Protein A as a capture step to effectively remove such impurities and byproducts. An effective polishing strategy is, however, irreplaceable, as > 1% of high molecular weight (HMW) species and > 100 ppm HCP often remains after the first Protein A capture step. In addition, further impurities such as leached protein A, potential increase in aggregates after viral inactivation steps or supplementary viral removal necessitate the presence of further polishing steps.

The common polishing approaches reported for bsAbs include separation techniques based on size, hydrophobicity, charge, or a combination of these methods otherwise known as multimodal chromatography. Besides size-based techniques which provide separation based on the hydrodynamic radius, many of the currently reported protocols are performed in the bind and elute mode, where the target molecule bound on the stationary phase is eluted via alterations often in the pH or salt concentration (Chen and Zhang 2021; Li et al. 2020). Charge-based purification is frequently employed in the form of ion exchange chromatography (Andrade et al. 2019; Jakobsen 2011; Vallera and Miller 2017; Guo et al. 2020; Allan et al. 2014; Brischwein et al. 2006; Kimerer et al. 2019; Sharkey et al. 2017; Igawa and Tsunoda 2009; Sampei et al. 2013; Hall et al. 2015), where bsAbs are commonly loaded in the absence of salt at a recommended pH of 1 to 3 units away from the isoelectric point (pI) of the target molecule followed by elution with buffer with high salt concentrations. Alternatively, the target molecule may be eluted with pH close to the target pI or with a combination of salt and pH effects. In contrast, hydrophobic interaction chromatography often makes use of a high concentration of kosmotropic salts such as ammonium sulfate to promote binding of the bsAb surface-exposed nonpolar residues to the hydrophobic ligands on the stationary phase, followed by elution at lower salt concentrations (Kimerer et al. 2020; Hall et al. 2018; Manzke et al. 1997; Fouque et al. 2016). By combining more than one fundamental separation technique in multimodal chromatography, the purification capabilities of existing purification platforms can potentially be further enhanced to obtain products of high purity and yield (Tustian et al. 2016; Guo et al. 2020; Fouque et al. 2016; Dimasi et al. 2019; Bertl et al. 2015; Tang et al. 2020).

In order to develop a robust process, 3 resins with different modes of separation—cation exchange, hydrophobic interaction and multimodal anion exchange resins—were selected for screening here. While size exclusion chromatography is a frequently reported polishing strategy for bsAbs (Jakobsen et al. 2011; Brischwein et al. 2006; Baehner et al. 2011; Bruenker et al. 2014; Geuijen et al. 2015; Kufer et al. 2014, 2007; Taylor et al. 2015; Pendzialek et al. 2017; Dorken et al. 2009; Spiesberger et al. 2015; Yang et al. 2015), it is not further explored here as their usage is mostly limited to purification processes at the laboratory scale due to low sample throughput. Capto S ImpAct and Capto Butyl ImpRes were selected as the former is a strong cation exchanger resin with a high binding capacity and high flow base matrix (Cytiva 2020a), whereas the latter is a hydrophobic resin with relatively high hydrophobicity (Cytiva 2020b). Capto adhere ImpRes was selected as the multimodal anion exchanger, as the hydrogen bonding and hydrophobic interactions in addition to ionic interactions may further enhance the purification capabilities depending on overall process conditions (Cytiva 2020c).

**Fig. 1:**
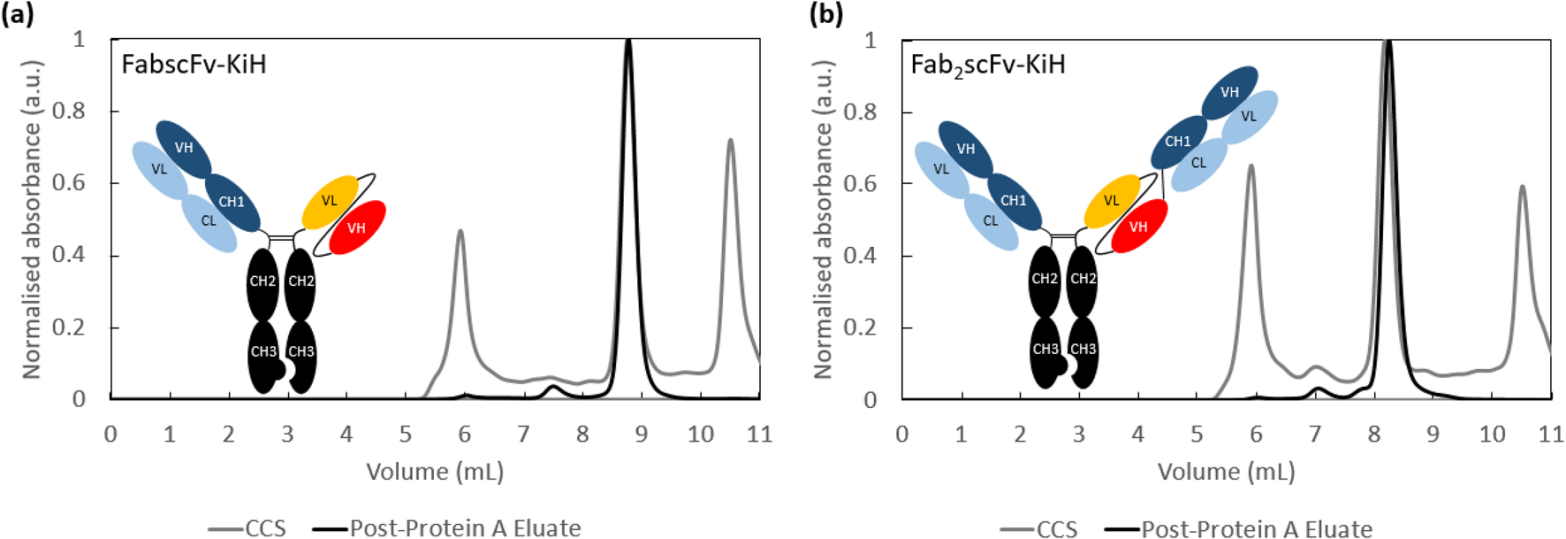
Schematic representation of model bsAbs—FabscFv-KiH (**a**) and Fab_2_scFv-KiH (**b**)—used in this study, along with their representative HPLC-SEC purity profiles of cell culture supernatant (CCS) and post-Protein A eluates

Here, using two knob-into-hole (KiH) bsAb post-Protein A eluates (Fig. 1, Table 1), we developed a two-step polishing process that can be utilised in the flow-through mode with effective removal of both HMW and HCP remaining in the post-Protein A eluate. Guided by the optimal conditions obtained from Design of Experiments (DoE) screening of three different resins with fundamentally different modes of separation mechanisms, we observed strong on-column aggregation of bsAbs on the Capto S ImpAct in the bind and elute mode, whereas Capto Butyl ImpRes and Capto adhere/Capto adhere ImpRes were effective flow-through polishing resins when employed as the first and second polishing steps, respectively, achieving a 17- to 35-fold HCP reduction and ~ 4–6% removal in HMW species with respect to monomer. Together with a previously optimised Protein A step, we demonstrate the effective purification of two KiH bsAb products consisting of < 1% HMW species, < 1% LMW species and < 100 ppm HCP, with an overall process recovery of 56–87%.

**Table 1:** Representative purity profile of cell culture supernatant (CCS) and post-Protein A eluates of FabscFv-KiH and Fab_2_scFv-KiH

		Load	Monomer concentration (mg/mL)	HCP (ppm)	Purity (%)		
					HMW	Mono	LMW
FabscFv-KiH	CCS	–	0.69	1,357,822	30.8	35.5	33.8
	Post-Protein A eluate	31.5 mg/mL-R	5.00	1779	5.7	93.9	0.4
Fab_2_scFv-KiH	CCS	–	0.72	1,329,885	33.5	31.1	35.4
	Post-Protein A eluate pH 4.3 wash	30.5 mg/mL-R	2.74	1551	6.1	92.1	1.8
	Post-Protein A eluate pH 4.1 wash		2.92	1526	6.5	92.1	1.4

## Materials and methods

Unless otherwise stated, all buffers, salts and reagents were purchased from Merck Millipore. All resins, PreDictor plates and Tricorn™ series columns (Cytiva) were kindly provided by Cytiva. bsAb culture and post-Protein A eluates were obtained as described previously (Chen et al. 2022).

### 96-well plate screening—determination of optimal conditions for column studies

For both stability and 96-well plate study, post-Protein A eluate (pH 6, 50 mM Na-citrate, no salt) was concentrated to 20 mg/mL using Amicon Ultra 15-mL filters (50 kDa NMWL), followed by adjustments to the required bsAb, pH and salt concentration.

The study was performed in 96-well plates, with data analysis performed using MODDE^®^ 12.1 software. 2-µL PreDictor Capto S ImpAct plate, 6-µL PreDictor Capto adhere ImpRes plates and 6-µL Capto Butyl ImpRes resin self-filled 96-well plates were used. All resins were equilibrated with the respective equilibration buffer (200 µL × 2). Post-Protein A eluates at the required pH and conductivities were then added to each well and incubated under shaking conditions (200 µL, 1 h, room temperature, 1100 rpm). The flow-through was collected and resin was washed once (200 µL). All removal of solution from each well was performed by centrifugation (300 g, 1 min). Both flow-through and wash fractions were combined and analysed by HPLC-SEC. Static binding capability (SBC) for Capto S ImpAct was determined using the following equation: $SBC=\frac{{load}_{mono}-{FT}_{mono}-{wash}_{mono}}{resin volume}$. Recovery for Capto Butyl ImpRes and Capto adhere ImpRes was calculated using the following equation: $Recovery=\frac{{FT}_{mono}+{wash}_{mono}}{{load}_{mono}}$.

### AKTA™ chromatography

All purification chromatography was conducted on an AKTA™ Avant 25 (Cytiva). 1 mL and 5 mL of the respective resins was packed in Tricorn™ series columns (Cytiva) with a bed height of 5.1 cm and 6.4 cm, respectively.

Capto Butyl ImpRes was equilibrated with 50 mM Na-citrate, pH 4.0, before loading the appropriate amount of sample. A 20 column volume (CV) wash with the same equilibration buffer was applied and flow-through > 50 mAu was collected for analysis. Capto adhere was equilibrated with 50 mM Na-citrate, pH 6.5 or pH 6.8, before loading the appropriate amount of sample. A 25 CV wash at the same pH was applied and both the flow-through during loading and 25 CV wash were collected. Capto S ImpAct was equilibrated with 20 mM citric acid and 20 mM sodium phosphate, pH 5.5, 50 mM NaCl, before loading the appropriate amount of sample. A 3 CV wash of equilibration buffer was performed followed by a linear gradient from equilibration buffer to the same buffer with 500 mM NaCl in 20 CV, with a 5 CV hold at the end. 4 min residence time was utilised for all polishing resins.

### Antibody concentration and purity analysis

HPLC-SEC was used to determine antibody concentration and purity, using a TSK_gel_ G3000SW_XL_ column (7.8 mm i.d. x 30 cm; Tosoh Bioscience). 100 µL of sample was injected for analysis, utilising a flow rate of 0.6 mL/min and a mobile phase which consisted of 0.2 M L-arginine, 0.05 M MES, 5 mM EDTA, 0.05% sodium azide (w/w), pH 6.5. The resultant concentrations were obtained by comparing the area under the peaks obtained at UV absorbance 280 nm with that of a calibration curve obtained using standard samples. The relative amount of HMW and LMW species was calculated based on the area of elution peaks before and after the monomeric peak, respectively. The area of the respective species obtained from HPLC-SEC was multiplied with the respective volume obtained from the AKTA system in order to perform mass balance analysis. Non-reducing SDS-PAGE gels (4–15% Criterion™ TGX Stain-Free™ Protein Gel, Bio-rad) were used according to manufacturer’s instructions, as a complementary approach to investigate the purity of the samples. Staining was performed with eLuminol™ (GeneCopoeia), with a total protein amount of 0.3 µg loaded per lane, as determined using Bradford assay (Thermo Fisher Scientific).

### Residual HCP and DNA analysis

Amersham HCPQuant CHO kit (Cytiva) was used according to manufacturer’s instructions to determine the CHO HCP content, with data acquisition performed on the Synergy™ 2 plate reader (BioTek).

A QX200™ Droplet Digital™ PCR System (Bio-Rad Laboratories) was used according to manufacturer’s instructions to measure the CHO DNA content. Briefly, samples were digested with proteinase K (0.2 mg/mL in 0.5% SDS, 16 h, 50 °C), followed by inactivation (10 min, 95 °C) and DNA extraction using QIAamp^®^ viral RNA mini kit (Qiagen). ddPCR™ supermix for residual DNA quantification (Bio-Rad Laboratories), ddPCR™ CHO residual DNA quantification assay (Bio-Rad Laboratories), Xeno™ VIC™ primer probe mix (Applied Biosystems), Xeno™ DNA control (Applied Biosystems) and the extracted DNA sample were then added together prior to droplet generation. The generation of droplets in a 96-well PCR plate was subsequently performed using an automated droplet generator (Bio-Rad Laboratories). All plates were heat-sealed with PX1™ PCR plate sealer (Bio-Rad Laboratories). A C1000 Touch™ thermal cycler (Bio-Rad Laboratories) was utilised for the PCR reaction (10 min at 95 °C; 40 cycles of 30 s at 94 °C followed by 1 min at 60 °C; 10 min at 98 °C). Subsequent data measurement and analysis was performed on the QuantaSoft analysis software (Bio-Rad Laboratories), with the conversion of DNA copy number to DNA concentration based on CHO host cell DNA standards (Applied Biosystems).

## Results

### Stability study and 96-well plate screening of optimal conditions for both bsAbs

**Fig. 2:**
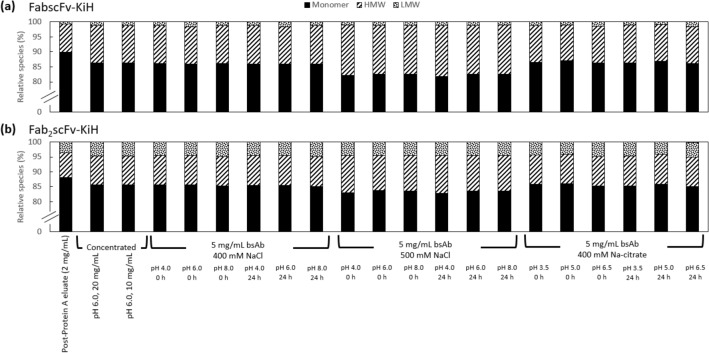
Stability study of FabscFv-KiH (**a**) and Fab_2_scFv-KiH (**b**) at the various bsAb, pH and salt concentrations

In order to establish the suitable range of screening conditions for subsequent polishing steps for both bsAbs, a stability study was first performed for both FabscFv-KiH and Fab_2_scFv-KiH (Fig. 2). Starting with a 2 mg/mL sample with monomer purity of 88–90% for both molecules, it was observed that the monomer purity decreased by ~ 3% upon concentrating to 20 mg/mL. This purity was maintained upon subsequent dilution to 10 and 5 mg/mL. A pH range from 4.0 – 8.0 and sodium chloride (NaCl) concentration up to 500 mM NaCl was subsequently investigated at 5 mg/mL bsAb concentration. While both bsAbs remained stable at 400 mM NaCl between pH 4.0 – 8.0, it was observed that the HMW species increased further by ~ 4% at 500 mM NaCl within the same range of pH values. The stability of both molecules was also confirmed in 400 mM Na-citrate between pH 3.5–6.5, as these are the potential pH and kosmotropic salt to be utilised for Capto Butyl ImpRes. No significant difference was observed at 0 h and 24 h of measuring the same sample after pH and salt adjustments. Based on these findings, a 96-well plate study was designed using this same batch of post-Protein A eluate (Table 2), taking into consideration that the pI of FabscFv-KiH and Fab_2_scFv-KiH is 8.5 and 8.6, respectively, and 400 mM is the highest salt concentration in which both bsAbs remained stable.

**Table 2:** 96-well plate screening conditions of Capto Butyl ImpRes, Capto adhere ImpRes and Capto S ImpAct for both FabscFv-KiH and Fab_2_scFv-KiH

	Buffer	pH	Salt
Capto Butyl ImpRes	Sodium citrate	4.0, 5.25, 6.5	20, 100, 200, 300, 400 mM Na-citrate
Capto adhere ImpRes	20 mM sodium citrate + 20 mM sodium phosphate	5.0, 5.75, 6.5	0, 50, 100, 150, 200, 250 mM NaCl
Capto S ImpAct	20 mM sodium citrate (pH 5.0–6.0) 20 mM sodium phosphate (pH 6.5–7.5)	5.0, 5.5, 6.0, 6.5, 7.0, 7.5	0, 50, 100 mM NaCl

For the selected polishing resins, Capto Butyl ImpRes is a hydrophobic resin, Capto adhere ImpRes is a multimodal anion exchanger with ionic interactions, hydrogen bonding and hydrophobic interactions, while Capto S ImpAct is a strong cation exchanger. Based on the above stability study results, only Capto S ImpAct is feasible to be explored as bind–elute mode polishing, as both Capto Butyl ImpRes and Capto adhere ImpRes require relatively high-salt conditions beyond the stable range of both bsAb molecules used in this study for bind–elute mode. Therefore, we decided to explore flowth-rough mode polishing instead for Capto Butyl ImpRes and Capto adhere ImpRes.

**Fig. 3:**
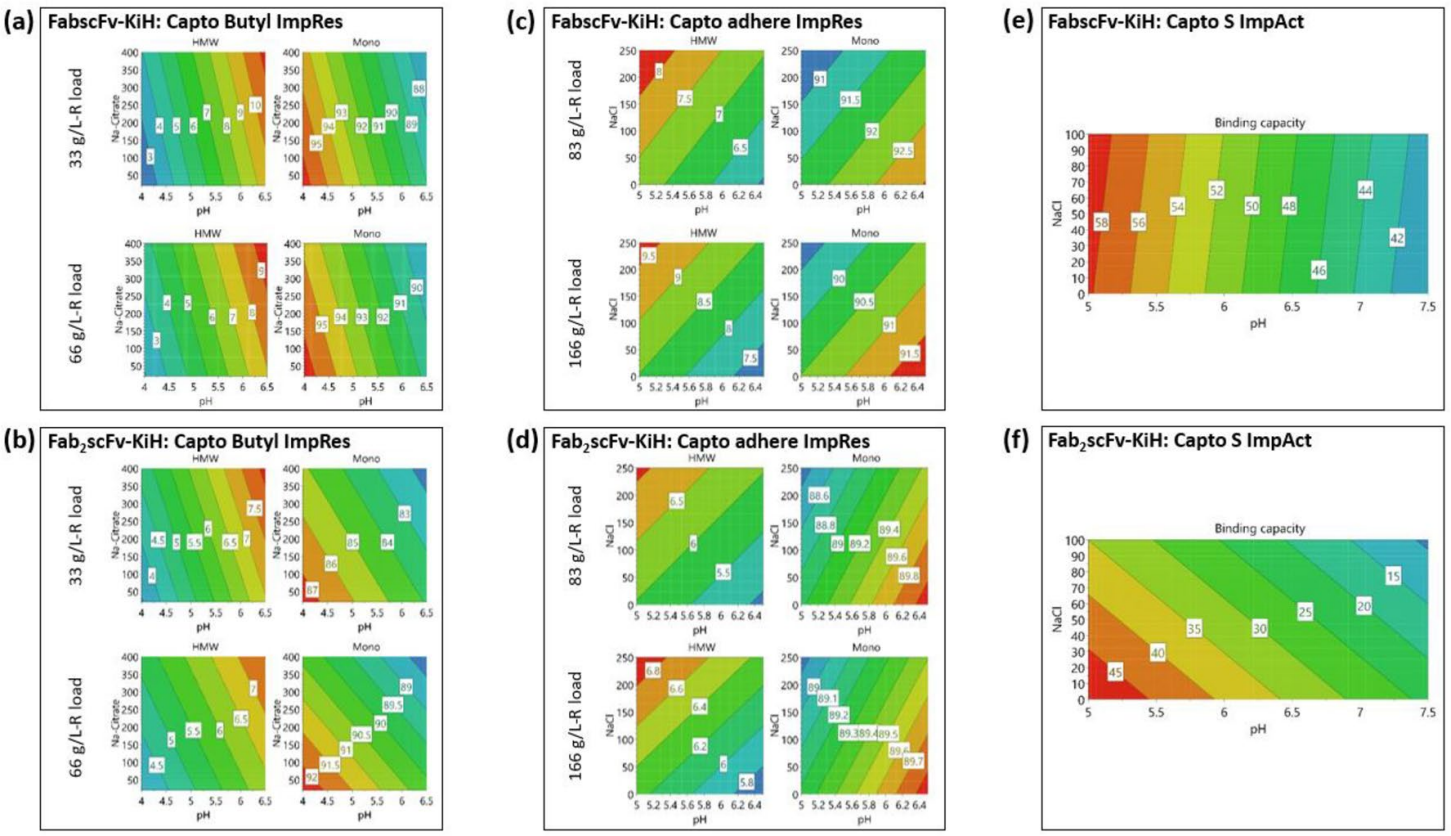
Contour plots obtained from DoE software after screening in 96-well plate format of Capto Butyl ImpRes (**a**, **b**), Capto adhere ImpRes (**c**, **d**), Capto S ImpAct resins (**e**, **f**) for both FabscFv-KiH and Fab_2_scFv-KiH

A screening study of flow-through conditions for Capto Butyl ImpRes was first performed between pH 4.0–6.5 and 20 mM–400 mM Na-citrate concentrations at 33 g/L-resin (R) and 66 g/L-R loads. As illustrated in the contour plots generated by using a DoE software (Fig. 3a, b), the highest monomer purity with lowest HMW species was obtained at pH 4 and low Na-citrate concentration for both molecules. The optimal flow-through condition for both molecules was therefore determined as 50 mM Na-citrate, pH 4.0 since the post-Protein A eluate contained 50 mM Na-citrate. The possibility of utilising the multimodal anion exchange resin, Capto adhere ImpRes, in the flow-through mode was also explored using conditions between pH 5.0–6.5 and 0–250 mM NaCl at 83 g/L-R and 166 g/L-R loads. As high monomer purity and low HMW species can be obtained at high pH (pH 6.5) with no NaCl for both molecules (Fig. 3c, d), this condition was selected for subsequent validation runs.

The optimal loading conditions for Capto S ImpAct was evaluated in the bind and elute mode by a screening study between pH 5.0–7.5 and 0–100 mM NaCl. The highest SBC was obtained at low pH, with 0–100 mM NaCl having no difference for FabscFv-KiH and no NaCl preferred for Fab_2_scFv-KiH. The optimal loading condition was therefore determined to be pH 5.0, with no salt concentration for both molecules. The maximum SBC determined in this way is 58 g/L-R or 45 g/L-R for FabscFv-KiH and Fab_2_scFv-KiH, respectively (Fig. 3e, f).

As Capto Butyl ImpRes demonstrated good HMW species removal with the lowest optimal pH condition of pH 4.0, which allows for minimal sample adjustment from pH 3.6 in the post-Protein A eluate, it stands out as a good choice of resin for the first polishing step. Capto adhere ImpRes and Capto S ImpAct were both evaluated as potential resins for the second polishing step, with the former allowing for a complete flow-through polishing process and potential virus removal capability, while the latter will yield a more concentrated final product due to its utilisation in the bind and elute mode.

### Evaluation of potential resins for first and second polishing steps in column format

**Fig. 4.**
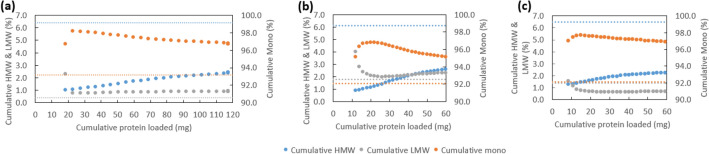
1-mL column validation runs of Capto Butyl ImpRes at the optimal condition of pH 4.0 for FabscFv-KiH (**a**) as well as Fab_2_scFv-KiH post-Protein A eluate with pH 4.3 wash (**b**) and pH 4.1 wash (**c**). The cumulative HMW (blue markers), LMW (grey markers) and monomeric (orange markers) species in the flow-through are represented as a function of the amount of bsAb loaded, with the initial percentage of each species represented in their respective colours in dotted lines

1-mL column validation runs were subsequently performed using bsAb post-Protein A eluates with purities similar to that reported in our previous study (Chen et al. 2022), based on the above determined optimised conditions. By loading up to 120 mg/mL and 60 mg/mL of FabscFv-KiH and Fab_2_scFv-KiH post-Protein A eluates on Capto Butyl ImpRes, respectively, it was observed that the HMW species can be reduced to ~ 2.5% in all cases (Fig. 4). LMW species was reduced to ~ 2% for Fab_2_scFv-KiH post-Protein A eluate with pH 4.3 wash (Fig. 4b) and ~ 1% for both Fab_2_scFv-KiH post-Protein A eluate with pH 4.1 wash and FabscFv-KiH post-Protein A eluate (Fig. 4a, c). The excellent HMW species removal observed in the 96-well plate format is validated here in 1-mL column format at high loading capacity, reaffirming the use of Capto Butyl ImpRes as the first polishing step.

**Fig. 5:**
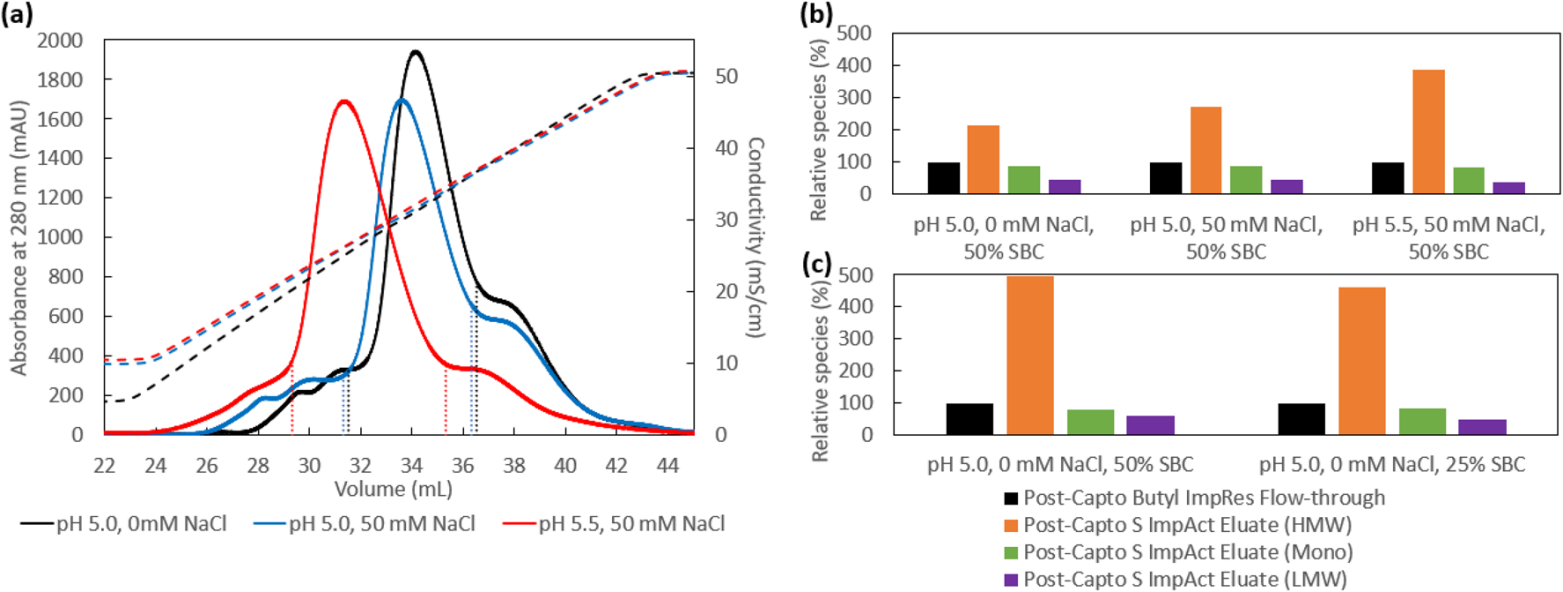
AKTA chromatogram elution profile of Capto S ImpAct (**a**), with UV_280nm_ and conductivity signals represented in solid and dotted lines, respectively. The HMW, monomer and LMW species present in the post-Capto S ImpAct eluate relative to those in the Capto Butyl ImpRes flow-through obtained at varying pH and NaCl conditions (**b**) and different load amounts (**c**) are illustrated

To evaluate the potential of Capto S ImpAct as a second polishing step, FabscFv-KiH post-Capto Butyl ImpRes flow-through was loaded onto a 1-mL column at a load corresponding to 50% of SBC, which corresponded to 29 g/L-R, at the conditions found in PreDictor plates of pH 5.0, 0 mM NaCl, followed by a 0–0.5 M NaCl 20 CV gradient elution (Fig. 5a). However, the amount of HMW species present in the entire post-Capto S ImpAct eluate increased by ~ twofold while the monomer and LMW species decreased compared to that present in the post-Capto Butyl ImpRes flow-through (Fig. 5b), suggesting the presence of significant aggregation during the bind/elute process. This is further supported by analysis of the early, mid- and late-fractions of the post-Capto S ImpAct eluate peak, which clearly shows an increase in the HMW species in the mid- and late-fractions (Table 3), with an overall low recovery of 88.9%. In order to investigate the effect of higher NaCl concentration and pH, post-Capto Butyl ImpRes flow-through was loaded at the same 50% SBC at pH 5.0, 50 mM NaCl and pH 5.5, 50 mM NaCl (Fig. 5a), followed by a 50–500 mM NaCl 20 CV gradient elution. It was observed that a higher salt concentration and a higher pH resulted in higher HMW species, along with a concomitant decrease in monomer and LMW species (Table 3, Fig. 5b), suggesting that a further reduction in overall charges and masking of electrostatic interactions can lead to greater on-column aggregation. In an attempt to reduce the amount of on-column aggregation, load was decreased to 25% of SBC. Although this resulted in a slight reduction of the HMW species, the on-column aggregation was still extremely significantly (Fig. 5c), pointing to the hypothesis that, for this bsAb which is prone to aggregate at higher concentration, bind and elute mode using a high-capacity resin like Capto S ImpAct may be less advantageous.

**Table 3:** Analysis of the monomer recovery and purity profile of post-Capto S ImpAct eluate in comparison to the load of post-Capto Butyl ImpRes flow-through reflects on-column aggregation of FabscFv-KiH on Capto S ImpAct

FabscFv-KiH		Monomer concentration (mg/mL)	Monomer recovery (%)	Purity (%)		
				HMW	Mono	LMW
Load (Post-Capto Butyl ImpRes flow-through)		1.76	–	2.7	96.3	1.0
Post-Capto S ImpAct: pH 5.0, 0 mM NaCl	Early fractions	0.22	4.5	1.5	62.2	36.3
	Mid fractions	3.56	61.3	5.9	93.7	0.3
	Late fractions	0.74	23.1	9.5	90.0	0.6
Load (Post-Capto Butyl ImpRes flow-through)		1.68	–	2.9	95.9	1.2
Post-Capto S ImpAct: pH 5.0, 50 mM NaCl	Early fractions	0.32	6.6	1.5	66.6	31.9
	Mid fractions	3.35	57.7	4.6	95.0	0.4
	Late fractions	0.59	20.3	13.8	85.7	0.5
Load (Post-Capto Butyl ImpRes flow-through)		1.68	–	2.9	95.6	1.5
Post-Capto S ImpAct: pH 5.5, 50 mM NaCl	Early fractions	0.4	1.4	1.6	89.3	9.1
	Mid fractions	3.33	67.0	3.6	95.8	0.6
	Late fractions	0.31	7.5	47.5	51.2	1.4

In comparison, Capto adhere ImpRes presents itself as a promising resin that can be utilised as the second polishing step in the flow-through mode in order to avoid on-column aggregation issues. While both Capto adhere and Capto adhere ImpRes resins have the same type of ligand and similar ligand density as well, the former has a larger particle size (75 µm for Capto adhere, 40 µm for Capto adhere ImpRes) and may be more suitable for flow-through mode applications (Cytiva 2020c, 2020d). We therefore next set out to evaluate different loading amounts on Capto Butyl ImpRes, the product of which can then be subsequently applied onto the Capto adhere resin, both employed in flow-through modes.

### Development of optimal flow-through polishing process with Capto Butyl ImpRes and Capto adhere as first and second polishing steps, respectively

Three additional loads of 20, 40 and 75 mg/mL-R of FabscFv-KiH post-Protein A eluate was loaded onto Capto Butyl ImpRes, which would correspond to an expected ~ 1%, 1.5%, 2% of HMW species obtained in the flow-through based on Fig. 4. It was observed that while 20 mg/mL-R load achieved < 1.0% HMW species, HCP remained more than 100 ppm (Table 4). As double the load yielded similar HCP levels with just a slight increase of 0.5% HMW species, 40 mg/mL-R was selected as the optimal load for the next polishing step. Using Capto adhere as the 2^nd^ polishing step at the conditions found in screening plates of pH 6.5, it was observed that < 1% HMW species can be achieved, although the HCP remained > 100 ppm (Table 5). To achieve a higher HCP removal efficiency, the effect of a slightly higher pH of 6.8 was investigated and it was observed that < 100 ppm can indeed be achieved under this condition, at the slight expense of recovery (Table 5).

**Table 4:** Effect of different load amounts on Capto Butyl ImpRes

Capto Butyl ImpRes, pH 4.0	Load	Monomer concentration (mg/mL)	Monomer recovery (%)	HCP (ppm)	Purity (%)		
					HMW	Mono	LMW
FabscFv-KiH	20 mg/mL-R	0.93	81.1	235	0.8	98.3	0.9
	40 mg/mL-R	1.34	90.8	243	1.3	98.0	0.7
	75 mg/mL-R	1.54	94.4	1114	2.1	97.4	0.6
	117 mg/mL-R	1.78	97.1	1713	2.7	96.4	0.9
Fab_2_scFv-KiH (Post-Protein A eluate with pH 4.1 wash)	15 mg/mL-R	0.98	78.4	144	1.5	96.7	1.7
	35 mg/mL-R	1.51	90.6	164	2.3	96.9	0.8
	60 mg/mL-R	1.67	90.4	345	2.4	96.9	0.7

**Table 5:** Comparison between the effect of pH 6.5 and pH 6.8 on Capto adhere flow-through mode using FabscFv-KiH

	FabscFv-KiH	Load	Monomer concentration (mg/mL)	Step monomer recovery (%)	Overall monomer recovery (%)	HCP (ppm)	Purity (%)		
							HMW	Mono	LMW
	CCS	-	0.69	-	-	1,357,822	30.8	35.5	33.8
Process 1	Post-Protein A eluate	31.5 mg/mL-R	5.00	91.1	91.1	1779	5.7	93.9	0.4
	Polishing FT mode: Capto Butyl ImpRes, pH 4.0	40 mg/mL-R	1.26	88.2	80.4	180	1.7	98.1	0.2
	Polishing FT mode: Capto adhere, *pH 6.5*	30 mg/mL-R	0.50	83.3	66.9	135	0.5	99.3	0.2
Process 2	Post-Protein A eluate	31.5 mg/mL-R	5.00	91.1	91.1	1779	5.7	93.9	0.4
	Polishing FT mode: Capto Butyl ImpRes, pH 4.0	40 mg/mL-R	1.26	88.2	80.4	180	1.7	98.1	0.2
	Polishing FT mode: Capto adhere, *pH 6.8*	30 mg/mL-R	0.45	75.0	60.3	81	0.4	99.4	0.2

For Fab_2_scFv-KiH, post-Protein A eluates obtained with a washing buffer of 50 mM Na-citrate, pH 4.3 or 4.1, respectively, before elution with 50 mM Na-citrate, pH 3.6 buffer were evaluated with the same polishing process parameters at a low loading of 20 mg/mL-R on Capto Butyl ImpRes, which corresponds to an expected ~ 1% HMW species in the flow-through for the pH 4.3 wash post-Protein A eluate, followed by a 15 mg/mL-R load on Capto adhere. The final product starting with the post-Protein A eluate with pH 4.3 wash yielded 101 ppm HCP and 4.0% LMW species, whereas that which started with the post-Protein A eluate with pH 4.1 wash consisted of 61 ppm HCP and 1.2% LMW species. As the use of the pH 4.1 wash post-Protein A eluate demonstrated superior final LMW species and HCP amounts in the final product, it was used for Capto Butyl ImpRes validation runs at 2 additional load amounts—15 and 35 mg/mL-R, corresponding to an expected ~ 1.5% and 2% of HMW species, respectively (Table 4). Using a pH 6.8 flow-through condition in Capto adhere, it was observed that all loading amounts led to < 100 ppm HCP and < 1% HMW species after 2 polishing steps, with higher loads resulting in higher recoveries and higher amounts of HCP present in the final products (Table 6).

**Table 6:** Evaluation of different load amounts on polishing steps for Fab_2_scFv-KiH

	Fab_2_scFv-KiH	Load	Monomer concentration (mg/mL)	Step monomer recovery (%)	Overall monomer recovery (%)	HCP (ppm)	Purity (%)		
							HMW	Mono	LMW
	CCS	-	0.72	–	–	1,329,885	33.5	31.1	35.4
Process 1	Post-Protein A eluate *(pH 4.1 wash)*	30.5 mg/mL-R	2.92	78.4	78.4	1526	6.5	92.1	1.4
	Polishing FT mode: Capto Butyl ImpRes, pH 4.0	15 mg/mL-R	0.98	78.4	61.5	144	1.5	96.7	1.7
	Polishing FT mode: Capto adhere, pH 6.8	10 mg/mL-R	0.16	56.0	34.4	57	0.0	97.8	2.1
Process 2	Post-Protein A eluate *(pH 4.1 wash)*	30.5 mg/mL-R	2.92	78.4	78.4	1526	6.5	92.1	1.4
	Polishing FT mode: Capto Butyl ImpRes, pH 4.0	35 mg/mL-R	1.51	90.6	71.0	164	2.3	96.9	0.8
	Polishing FT mode: Capto adhere, pH 6.8	30 mg/mL-R	0.49	75.1	53.3	84	0.2	98.9	0.9
Process 3	Post-Protein A eluate *(pH 4.1 wash)*	30.5 mg/mL-R	2.92	78.4	78.4	1526	6.5	92.1	1.4
	Polishing FT mode: Capto Butyl ImpRes, pH 4.0	60 mg/mL-R	1.67	90.4	70.9	345	2.4	96.9	0.7
	Polishing FT mode: Capto adhere, pH 6.8	45 mg/mL-R	0.65	79.4	56.3	88	0.3	98.6	1.1

**Fig. 6:**
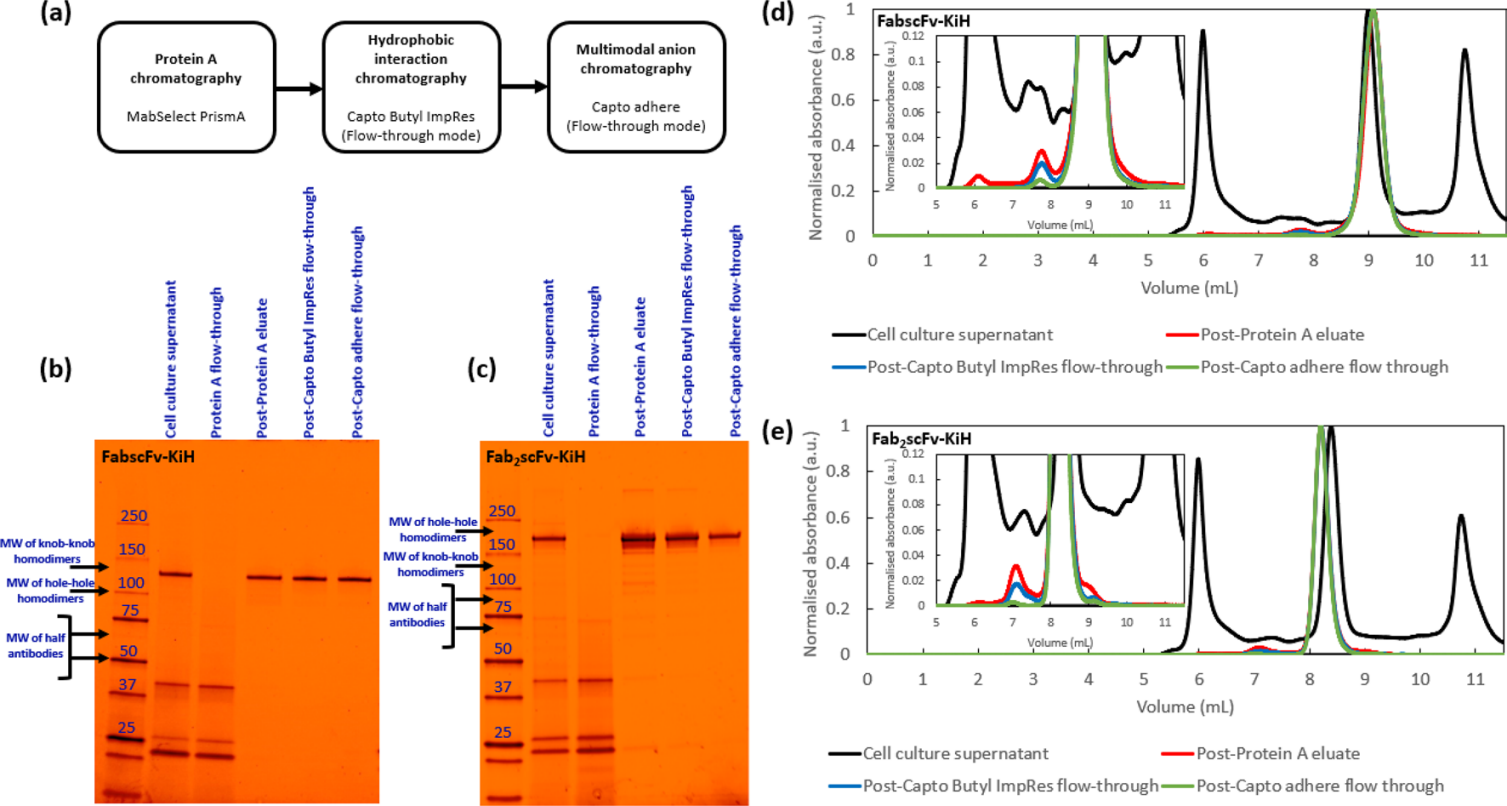
The schematic purification strategy proposed for KiH bsAbs (**a**). SDS-page gels (**b**, **c**) and SEC-HPLC chromatograms with zoomed-in insets (**d**, **e**) illustrating the purity profiles for FabscFv-KiH (**b**, **d**) and Fab_2_scFv-KiH (**c**, **e**) obtained in this three-step purification process

The robustness of the process was finally validated in 5-mL columns using the highest load amount evaluated in the full process in 1-mL columns (Table 7). It was observed that the 5-mL column validation runs yield similar purity and higher overall recovery as compared to that of 1-mL column, with low amounts of leached Protein A detected in the post-Protein A eluates and low levels of host cell DNA present in all the products. The effectiveness of the full process (Fig. 6a) in producing a final product of high purity is further illustrated in the SDS-PAGE gels (Fig. 6b, c) and HPLC-SEC chromatograms (Fig. 6d, e), which clearly demonstrates the improvement in purity profile with each downstream processing step. Most half-antibodies and homodimers (especially hole–hole homodimers) for both FabscFv-KiH and Fab_2_scFv-KiH were removed by Protein A chromatography under optimised conditions, hence the major byproducts removed in the polishing steps were aggregates and HCP. Nevertheless, there were still a little half-antibody and homodimer byproducts remaining in the Protein A eluates for both bsAb molecules, which were further reduced after polishing.

**Table 7:** 5-mL validation runs of the fully optimised process for both FabscFv-KiH and Fab_2_scFv-KiH

		Load	Monomer concentration (mg/mL)	Step monomer recovery (%)	Overall monomer recovery (%)	Leached Protein A (ppm)	HCP (ppm)	hcDNA (ppm)	Purity (%)		
									HMW	Mono	LMW
	CCS	–	0.56	-	-	N.D	1,307,373	67,006.3	30.8	34.0	35.2
FabscFv-KiH	Post-Protein A eluate *(pH 4.7 wash)*	31.5 mg/mL-R	3.68	91.1	91.1	5	2350	0.5	4.8	94.7	0.5
	Polishing FT mode: Capto Butyl ImpRes, pH 4.0	40 mg/mL-R	2.25	92.7	84.4	N.D	189	0.5	1.8	98.1	0.1
	Polishing FT mode: Capto adhere, pH 6.8	30 mg/mL-R	0.76	88.7	74.9	N.D	87	0.2	0.6	99.3	0.1
	CCS	–	0.72	-	-	N.D	1,077,136	54,133.9	33.5	31.1	35.4
Fab_2_scFv-KiH	Post-Protein A eluate *(pH 4.1 wash)*	30.5 mg/mL-R	1.75	78.4	78.4	6	2064	0.5	5.1	93.2	1.7
	Polishing FT mode: Capto Butyl ImpRes, pH 4.0	60 mg/mL-R	1.52	93.0	72.9	N.D	153	0.3	2.6	96.6	0.8
	Polishing FT mode: Capto adhere, pH 6.8	45 mg/mL-R	0.73	82.1	59.9	N.D	59	0.1	0.3	99.2	0.5

Representative post-Protein A eluates are reported in the table, as multiple post-Protein A eluates from 5-mL column are pooled together to generate sufficient materials for the subsequent polishing steps

## Discussion

Bispecific antibodies, especially scFv containing molecules, have frequently been reported to possess increased aggregation propensities, (Garber 2014; Taki et al. 2015; Andrade et al. 2019; Michaelson et al. 2009; Michaelson et al. 2009; Jakobsen et al. 2011; Vallera and Miller 2017). Although legacy polishing strategies such as salt gradient or pH gradient-based ion exchange methods may still work for common light chain and CrossMAb format bsAbs, they may not be applicable to scFv containing bsAbs due to their higher aggregation propensity.

Here we report that the stability of the two model bsAbs—FabscFv-KiH and Fab_2_scFv-KiH—was maintained at 400 mM NaCl pH 4 – 8 and 400 mM Na-citrate pH 3.5–6.5 at 5 mg/mL bsAb concentration. The HMW species was, however, increased by 1–3% upon concentrating from 2 mg/mL to 20 mg/mL and further increased by ~ 4% at 500 mM NaCl between pH 4 – 8 at 5 mg/mL. Furthermore, the higher aggregation propensity of scFv containing bsAbs was also reflected in the on-column aggregation of the FabscFv-KiH in Capto S ImpAct, which was further exacerbated by the presence of higher salt concentrations and higher pH closer to the pI. The slight reduction in on-column aggregation at lower loading amounts is in line with our previous observation, where a reduced load on Protein A chromatography column resulted in reduced on-column aggregation effects (Chen et al. 2022).

The development of a complete flow-through polishing method for bsAbs at our proposed optimised conditions therefore presents several advantages. Considering the higher aggregation propensity of scFv containing bsAbs, a flow-through methodology circumvents on-column aggregation caused by an increase in local bsAb concentrations, hence preventing unwanted product loss. In addition, the need for additional elution buffer preparation and consumption is eliminated, increasing the overall ease of process operation. Furthermore, the optimised hydrophobic interaction chromatography process using Capto Butyl ImpRes in the flow-through mode developed here does not require the addition of high concentration of salts that is typically required in the bind and elute mode (Kimerer et al. 2020; Hall et al. 2018; Manzke et al. 1997; Fouque et al. 2016), thus further preventing potential salt-induced product aggregation and again increases the ease of process operation. In addition, the step-wise pH increment of the post-Protein A eluate from pH 3.6 to pH 4.0 and finally pH 6.8 requires minimum sample adjustments and allows for an additional low pH virus inactivation step to be introduced between the Protein A and Capto Butyl ImpRes step, with Capto adhere providing potential virus removal capabilities (Cytiva 2020d). Despite the above advantages of a complete flow-through polishing strategy, the final protein is very dilute compared to the product obtained via bind–elute polishing methods. Therefore, it will be necessary to concentrate the protein more folds during diafiltration step in order to make this a commercially viable workflow.

Although the HMW species was quantitatively very similar in both FabscFv-KiH and Fab_2_scFv-KiH post-Protein A eluates at ~ 6%, ~ 2.5% of HMW species was obtained when 120 mg/mL-R and 60 mg/mL-R of FabscFv-KiH and Fab_2_scFv-KiH post-Protein A eluates were loaded onto Capto Butyl ImpRes, respectively. The fact that the same relative percentage of HMW species can be yielded at double the load of FabscFv-KiH post-Protein A eluate compared to that of Fab_2_scFv-KiH reflects a higher binding capability of Capto Butyl ImpRes towards the HMW species present in the former compared to the latter. This highlights the importance of investigating the removal of different species as a function of different loading amounts, as the type and physicochemical properties of the exact species present in the sample, in addition to the quantity, plays an important role in determining the effectiveness of their removal.

In comparison to FabscFv-KiH, the Fab_2_scFv-KiH post-Protein A eluate contained a higher amount of LMW species, with the LMW species being ~ 1.8% for Fab_2_scFv-KiH post-Protein A eluate with pH 4.3 wash and ~ 1.4% for Fab_2_scFv-KiH post-Protein A eluate with pH 4.1 wash. By applying the same polishing strategy and process parameters on these two eluates, 4.0% and 1.2% final LMW species was obtained for Fab_2_scFv-KiH post-Protein A eluate with pH 4.3 and pH 4.1 wash, respectively. This demonstrates the importance of developing a robust process for each step of the purification methodology, as each plays an important role and may have important downstream effects.

## Conclusions

In conclusion, complementary to Protein A, polishing steps play a critical role in removing the remaining HMW and LMW species, as well as HCP in order to achieve a final product of high purity. We demonstrated here using two KiH bsAb post-Protein A eluates that Capto Butyl ImpRes and Capto adhere can result in ~ 4–6% removal of HMW species with respect to monomer and 17- to 35-fold reduction of HCP at 30–60 mg/mL load. Through the employment of these two resins in a complete flow-through mode after an optimised Protein A chromatography step, a final product containing < 1% HMW species and < 100 ppm HCP can be obtained with an overall process recovery of 56–87%. Such a flow-through polishing strategy prevents on-column aggregation and improves the overall ease of operation of the process without the need for additional elution buffer preparation and consumption, allowing for increased adaptability and alignment with process intensification efforts.

## Data Availability

All data generated or analysed during this study are included in this published article.

## Abbreviations

bsAbs: Bispecific antibodies
HMW: High molecular weight
LMW: Low molecular weight
HCP: Host cell protein
KiH: Knob-into-Hole
R: Resin
DoE: Design of Experiments
pI: Isoelectric point
SBC: Static binding capability
CV: Column volume
NaCl: Sodium chloride
FT: Flowthrough
DBC: Dynamic binding capacity
CCS: Cell culture supernatant
